# Challenges with two epidural catheters for labor analgesia in a patient with lumbar adhesions: a case report

**DOI:** 10.1186/s40981-024-00724-1

**Published:** 2024-06-18

**Authors:** Yuki Hosokawa, Rie Kato, Eriko Ohsugi, Michiko Sugita

**Affiliations:** 1 Department of Anesthesiology, Showa University School of Medicine, 1-5-8 Hatanodai, Shinagawa-Ku, Tokyo, 142-8555 Japan; 2 Department of Anesthesiology, Kumamoto University School of Medicine, 1-1-1 Honjo, Chuo-Ku, Kumamoto, 860-8556 Japan

**Keywords:** Epidural adhesion, Epidural labor analgesia, Double epidural catheter, Case report

## Abstract

**Background:**

The efficacy of neuraxial analgesia varies with spinal canal pathology. Notably, a secondary epidural catheter has been shown to increase neuraxial labor analgesia in women with spinal lesions. Therefore, we present a case in which catheter withdrawal played a critical role in achieving effective labor analgesia in a woman with epidural adhesions after lumbar discectomy who had inadequate analgesia with two epidural catheters.

**Case presentation:**

We encountered a patient with L5 lumbar epidural adhesions who reported pain even after receiving two epidural catheters. The catheters were placed in the L1/2 and L5/S intervertebral spaces. Analgesic effects were exerted when the L5/S catheter was withdrawn by 1 cm, suggesting that the catheter tip was initially placed inside the adhesion.

**Conclusions:**

Careful consideration of catheter placement and adjustments by withdrawing the catheter are crucial in managing labor analgesia in patients with known epidural adhesions.

## Background

The use of neuraxial blockade in patients with preexisting spinal stenosis, lumbar disk disease, or prior spinal surgery is controversial [1]. Furthermore, the efficacy of neuraxial analgesia with spinal canal pathology varies, with some reports showing a higher failure rate of analgesia and others showing comparable rates [1–3]. The spread of anesthetic solutions can be unpredictable in patients with lumbar epidural adhesions. Consequently, placing a second epidural catheter has been used to manage inadequate analgesia in women with spinal pathology [4, 5].

Therefore, we present a case in which catheter withdrawal played a critical role in achieving effective labor analgesia in a woman with epidural adhesions after lumbar discectomy who had inadequate analgesia with two epidural catheters.

### Case presentation

**Fig. 1:**
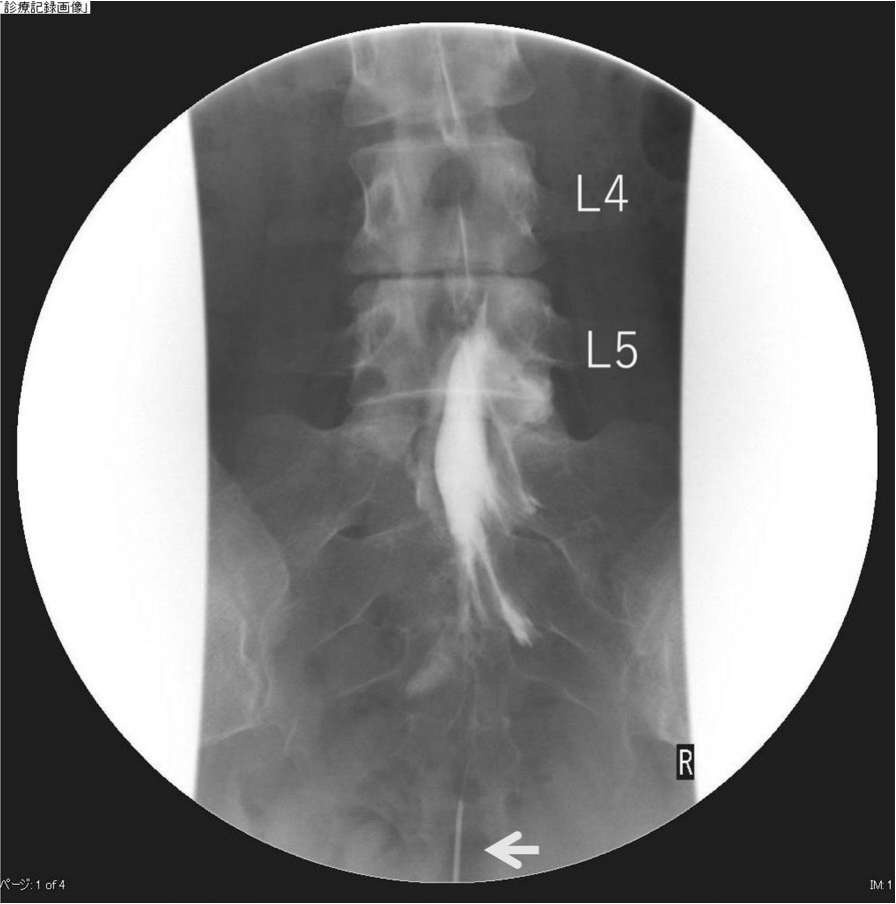
Epidurography 1 year before delivery. Epidurography showing a filling defect above the upper third of the L5 vertebra. The arrow shows a catheter inserted into the sacral hiatus for a contrast injection

A 29-year-old gravida 2 para 1 woman with a normal pregnancy presented for labor induction with neuraxial analgesia at 38 weeks and 4 days of gestation. She had not received epidural analgesia for her previous delivery. Her medical history included failed back surgery syndrome after L4/5 discectomy, with no neurologic deficits other than back pain. Epidurography conducted 1 year before the delivery showed an epidural adhesion cephalad to the upper one-third of the L5 vertebra (Fig. 1) and confirmed the absence of the sixth lumbar vertebra.

We predicted that local anesthetic solutions administered from L3/4, where we usually place epidural catheters for labor analgesia, would not spread to the sacral epidural space; therefore, we planned to place two epidural catheters: one cephalad (L1/2) and another caudal (L5/S) to the epidural adhesion. Moreover, a dural puncture epidural technique was planned for caudal epidural catheter placement to accelerate sacral analgesia onset.

**Fig. 2:**
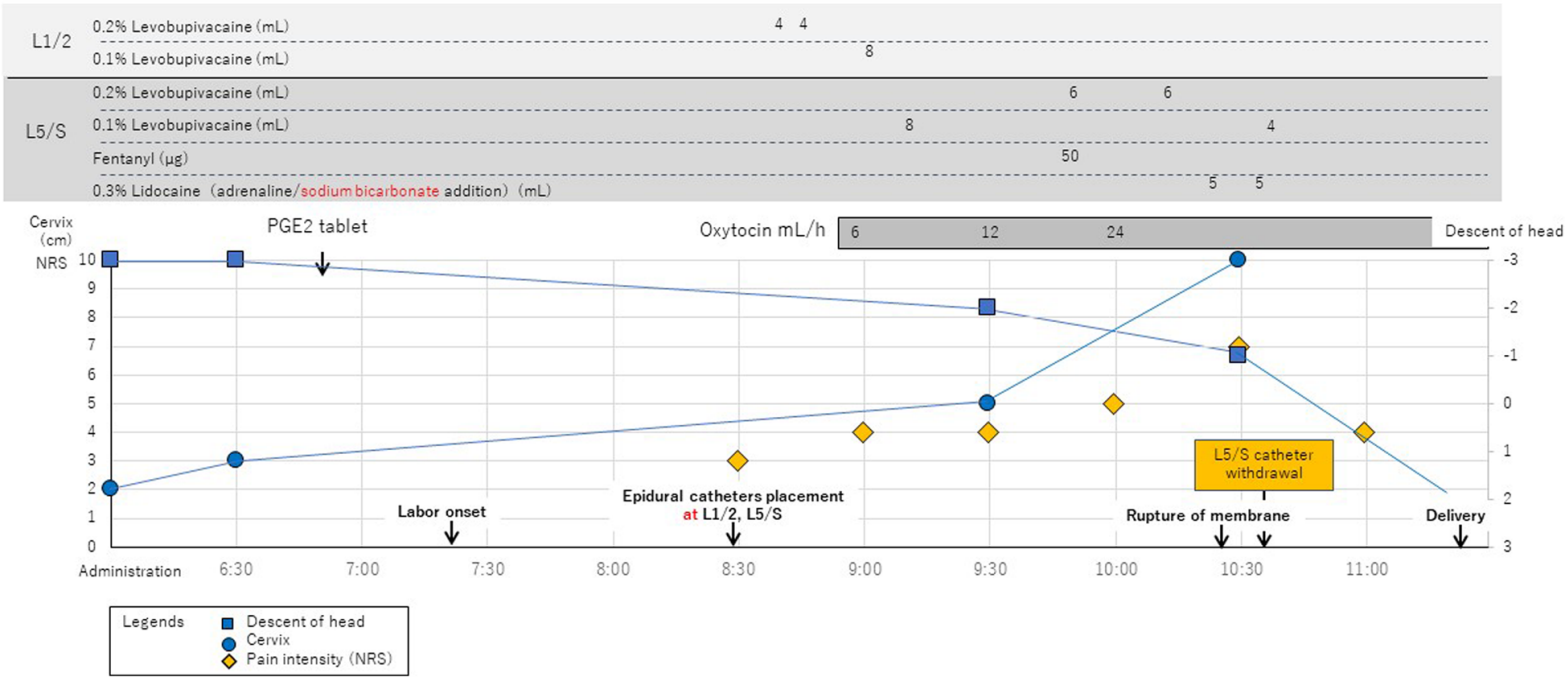
Labor and analgesic process

Labor was induced with oral prostaglandin E2 and intravenous oxytocin. After labor onset, two closed-end catheters with three lateral holes (Perifix™ SoftTip Catheter, B. Braun, Tokyo, Japan) were inserted 4 cm cephalad into the epidural space at the L1/2 and L5/S intervertebral spaces, respectively, while the patient was sitting. Intervertebral levels were identified using ultrasonography and counted from the sacrum. A dural puncture was performed at L5/S using a 27G pencil-point spinal needle (Portex™ Secure CSE needle, Smith Medical Japan, Tokyo, Japan). Figure 2 shows the labor progression, medication dosages, and cold sensitivity along dermatomes.

The patient reported mild pain (numerical rating scale (NRS): 3) 1 h and 20 min after labor onset. The patient did not request analgesics; however, we administered 8 mL of 0.2% and 0.1% levobupivacaine through the L1/2 catheter after confirming no blood or cerebrospinal fluid aspiration. Furthermore, 0.08% levobupivacaine with 2 µg/mL fentanyl was administered through programmed intermittent bolus (PIB) from the cephalic catheter after observing decreased cold sensitivity at the bilateral Th10 dermatome. Epidural infusion pump settings were 8-mL PIB every 60 min, starting 30 min after the initial epidural effect was confirmed, and 6 mL patient-controlled analgesic with a 10-min lockout interval.

The patient complained of moderate lower abdominal pain (NRS 4) 2 h after labor onset. Therefore, we administered 8 mL of 0.1% levobupivacaine via the L5/S catheter. As the pain increased (NRS 5) 2.5 h into labor, and the evaluation of cold discrimination revealed decreased cold sensitivity at Th10-L3 on the right and Th10–Th11 on the left dermatome, 6 mL of 0.2% levobupivacaine and 50-µg fentanyl were sequentially administered via the L5/S catheter.

**Fig. 3:**
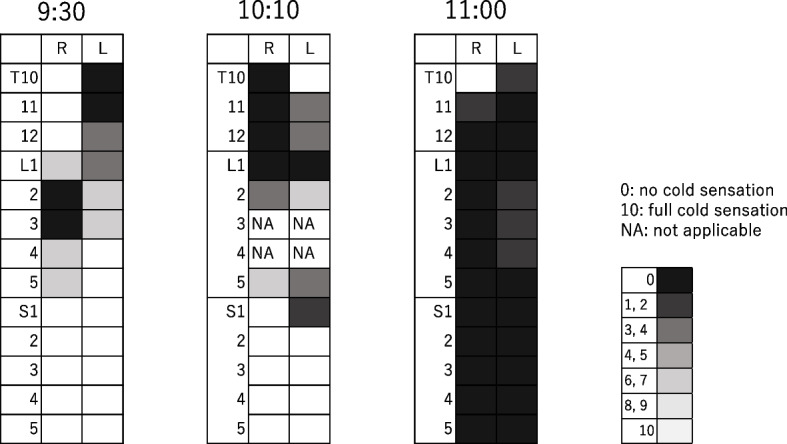
Evaluation of cold discrimination by NRS. NRS, numerical rating scale

However, she still complained of moderate pain (NRS 5) 3 h after labor onset. Evaluation of cold discrimination revealed cold sensitivity at Th10-L3 on the left and L1–L5 on the right dermatome (Fig. 3, 9:30). Cervical ripening and dilation suggested labor progression; therefore, 6 mL of 0.2% levobupivacaine was administered through the L5/S catheter. Rapid labor progression commenced after amniotic sac rupture and cervix dilation, and the patient reported severe pain (NRS 7). Therefore, we administered 5 mL of 0.3% lidocaine through the L5/S epidural catheter twice. Adrenaline and sodium bicarbonate were added to enhance the analgesic effect; however, no sensory blockade on the sacral segments was achieved (Fig. 3, 10:10). We retracted the L5/S catheter by 1 cm and administered 4 mL of 0.2% levobupivacaine, and this resulted in a marked improvement in analgesia (Fig. 3, 11:00).

Notably, 4 h and 5 min after the onset of labor, an infant was delivered with Apgar scores of 8 and 9 at 1 and 5 min, respectively. The patient did not report pain at delivery (NRS 0) and was satisfied with the analgesia during labor. She was discharged 5 days after delivery, following the standard course in our hospital, without any anesthetic complications.

## Discussion

In this case, two epidural catheters placed cephalad and caudal to an epidural adhesion initially failed to provide adequate sensory blockage. Withdrawing the caudal epidural catheter expanded the sensory block segments, subsequently achieving adequate analgesia.

The efficacy of neuraxial analgesia in patients with spinal canal pathology varies, with some reports showing a higher failure rate of analgesia and others showing comparable rates [1–3]. A second epidural catheter has been used to manage inadequate neuraxial labor in women with spinal lesions [5, 6]. Schachner et al. described a case of traumatic lumbar disc injury (L4/5), where an epidural catheter placed at L2/3 did not provide sensory block below L5 on the right; a secondary epidural catheter added to L4/5 provided analgesic effects in the unblocked segment [5]. Martinez et al. reported a case of previous lumbar fusion (L1–L4) due to a sports injury, in which an epidural catheter was placed at L4/5. However, the drug’s cephalad spread was insufficient, requiring an additional epidural catheter to be placed at Th12/L1 to improve analgesia [4]. In contrast to these two cases where inadequate analgesia was not predicted before labor, our case involved the preemptive use of two epidural catheters because the anesthetic solution was expected not to spread beyond the adhesion. However, this problem was not resolved with the use of two catheters.

In the present case, the analgesic effect on the sacral segments was insufficient despite two epidural catheters being placed cephalad and caudal to the adhesion site. This could be because the catheter tip was inserted into the adhesion, hindering local anesthetic diffusion. Epidurography showed that the contrast injected from the sacrum did not spread cephalad from the upper one-third of the L5 vertebral body, indicating epidural adhesion. The average height of lumbar vertebral bodies in Japanese women is approximately 29.0–30.0 mm [7]; therefore, the shortest distance from the yellow ligament to the caudal inferior border of the adhesion is approximately 2 cm. An epidural catheter with a closed tip was inserted 4 cm inside the epidural space, with three holes located at 0.5 cm, 1.0 cm, and 1.5 cm from the tip. If aligned straight, the adhesion site would have been 2 cm away from the tip, meaning all holes would have been within the adhesion. Typically, in multi-hole catheters, the solution exits primarily from the proximal hole, and the outflow is more distal with increasing flow rate [8]. In this case, we manually administered a local anesthetic through the L5/S catheter, indicating a high flow rate and equal outflow from the three holes. The anesthetic could have flowed out of the proximal hole into the adhesion, restricting the spread of the solution. The contrast from the catheter placed through the sacral hiatus spread below L5; therefore, a caudal epidural block may have been a better alternative for managing insufficient analgesia in the sacral region.

Although epidural catheter withdrawal is a common practice in obstetric anesthesia, its effectiveness specifically for breakthrough pain has not been proven [9]. Beilin et al. [10] compared catheter withdrawal followed by injection of local anesthetic with injection of local anesthetic without catheter withdrawal in managing breakthrough pain. They showed that the analgesic effect did not differ between the groups. However, in the present case, catheter withdrawal was highly effective. This indicates that epidural adhesions may inhibit the distribution of local anesthetic in the epidural space.

A limitation of this case was our inability to confirm the exact position of the epidural catheter. Since the catheter was radiolucent, it could not be confirmed using radiography; however, the position of the epidural catheter and the drug spread could have been verified using contrast imaging from both catheters after delivery.

In conclusion, while the initial placement of two epidural catheters did not provide sufficient analgesia, adjusting the position of the L5/S catheter by 1-cm withdrawal significantly improved analgesia. This suggests that careful consideration of catheter placement and subsequent adjustments are crucial in providing labor analgesia in patients with known epidural adhesions.

## Data Availability

Not applicable due to patient privacy concerns.

## Abbreviations

NRS: Numerical rating scale
PIB: Programmed intermittent bolus
